# Progressive Multifocal Leukoencephalopathy in AIDS: The Diagnostic Role of PET Imaging

**DOI:** 10.3390/idr18020033

**Published:** 2026-04-08

**Authors:** Virginia Donini, Riccardo Paggi, Alberto Farese, Costanza Malcontenti, Enrico Tagliaferri, Claudio Caroselli, Spartaco Sani, Maria Matteini, Alessandro Bartoloni, Lorenzo Zammarchi

**Affiliations:** 1 Department of Experimental and Clinical Medicine, University of Florence, 50134 Florence, Italy; virginia.donini@unifi.it (V.D.); alessandro.bartoloni@unifi.it (A.B.); 2 Infectious and Tropical Diseases Unit, Careggi University Hospital, 50134 Florence, Italy; paggir@aou-careggi.toscana.it (R.P.); faresea@aou-careggi.toscana.it (A.F.); malcontentic@aou-careggi.toscana.it (C.M.); 3 Infectious Diseases Unit, Livorno Hospital, 57124 Livorno, Italy; enrico.tagliaferri@uslnordovest.toscana.it (E.T.); claudio.caroselli@uslnordovest.toscana.it (C.C.); spartaco.sani@uslnordovest.toscana.it (S.S.); 4 Nuclear Medicine Unit, Careggi University Hospital, 50134 Florence, Italy; matteinim@aou-careggi.toscana.it

**Keywords:** progressive multifocal leukoencephalopathy, PML, AIDS, FDG-PET, PET

## Abstract

Introduction: The majority of progressive multifocal leukoencephalopathy (PML) cases is still represented by patients affected by acquired immunodeficiency syndrome (AIDS). Diagnosis of PML relies on histopathological findings or by the combination of clinical signs, radiological evidence, and molecular positivity of the JC virus in cerebrospinal fluid. However, AIDS status predisposes to various diseases involving the brain, testing the diagnostic ability of the clinician. Case description: We describe a PML case in a patient with AIDS, in whom lumbar puncture was initially impossible for severe thrombocytopenia and magnetic resonance showed an hyperintense lesion and was unable to distinguish between PML and lymphoma. In this case, [^18^F]-fluorodeoxyglucose (FDG)-PET imaging showing a hypometabolism of the lesion helped to initially orient toward PML, as diagnosis was later confirmed by lumbar puncture. We collected 21 cases in the literature in which [^18^F]-FDG-PET was helpful in cases of PML. Discussion and Conclusions: PET imaging is not considered a standard diagnostic tool for PML. However, in selected cases, it may provide valuable information to direct the diagnosis towards PML.

## 1. Introduction

Progressive multifocal leukoencephalopathy (PML) is an opportunistic infection of the central nervous system (CNS) caused by the polyomavirus JC (JCV). The virus is frequently contracted during childhood where it causes asymptomatic infections, then remains latent in the lungs, kidneys and reticulo-endothelial system. Under conditions of immunosuppression, the virus may reactivate, reaching the CNS, where it replicates within oligodendrocytes leading to cell death and progressive demyelination [1]. The current diagnostic criteria for definite PML are stringently based on the demonstration of JCV in brain tissue by biopsy or, in the context of a typical magnetic resonance imaging (MRI) and clinical presentation, the demonstration of JCV-DNA in the cerebrospinal fluid (CSF) [2,3].

Progressive multifocal leukoencephalopathy symptoms depend on the locations involved, but the most common include cognitive and behavioral abnormalities, sensory and motor deficits, ataxia, aphasia and cortical visual changes [4].

On brain MRI, the typical lesion is hyperintense on T2-weighted FLAIR sequences, involves subcortical and juxtacortical white matter, and usually (but not always) appears sharply delineated at the cortical border [5,6]. Brain MRI is also crucial for monitoring the disease; in patients with partial or complete immune reconstitution, PML lesions tend to expand slowly for weeks to months, after which PML immune reconstitution inflammatory syndrome (IRIS) may develop. In PML-IRIS, lesions undergo rapid expansion, accompanied by signs of inflammation (e.g., perilesional edema, mass effect, enlargement of perivascular spaces, and contrast enhancement) [1].

## 2. Case Description

**Figure 1:**
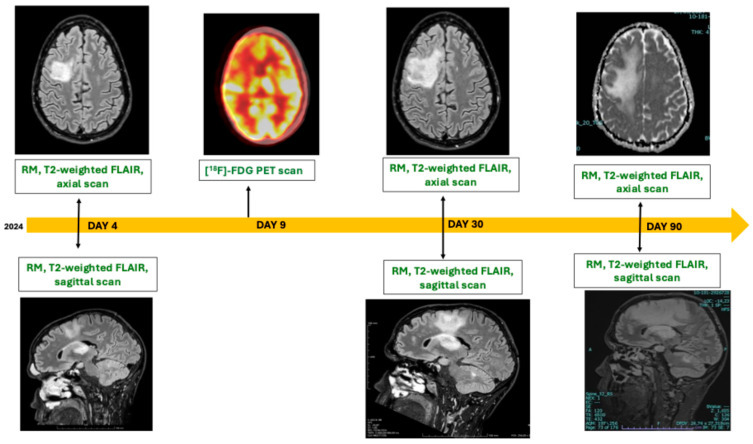
Timeline of encephalic radiological assessments performed in 2024. FLAIR: fluid-attenuated inversion recovery; [^18^F]-FDG-PET: fluorodeoxyglucose positron emission tomography; RM: magnetic resonance.

On 30 June 2024, a 29-year-old man went to the emergency department for persistent difficulty in concentration, weakness and onset of slurred speech for about 10 days. Blood tests showed pancytopenia characterized by severe thrombocytopenia (15 × 10^9^/L), anemia (hemoglobin 11.5 g/dL), and leukopenia (white blood cells 3.6 × 10^9^/L). Brain CT scan showed white matter hypodensity in the right nucleocapsular site with extension to the corona radiata, semioval center, and in subcortical white matter in the frontal site with preserved grooves. He was admitted to internal medicine. On day three after hospital admission (D3), he tested positive for HIV with HIV-RNA 3,700,000 cp/mL, CD4+ 130/mmc (5.6%), and CD4/CD8 ratio 0.1. On D5, the patient was admitted to the Infectious Diseases Department and on D6, antiretroviral therapy with bictegravir, emtricitabine, and tenofovir alafenamide fumarate was initiated. On D4 he underwent a brain MRI showing a lesion, hyperintense in T2 fluid-attenuated inversion recovery (FLAIR) and hypointense in T1 sequences, in the right frontal subcortical white matter descending caudally through the ipsilateral capsular–lenticular region up to the level of the anterior thalamus. Other areas of abnormally hyperintense signal in T2/FLAIR sequence in bihemispheric subcortical location and in left parietal cortico-subcortical location adjacent to the corpus callosum were documented (Figure 1). The report, in agreement with the neuroradiologist, did not allow us to distinguish between PML and lymphoma; both diagnoses were therefore possible, but considering thrombocytopenia, the patient had a contraindication to lumbar puncture. In order to differentiate the diagnosis and better characterize the lesions, a [^18^F]-fluorodeoxyglucose (FDG) positron emission tomography scan ([^18^F]-FDG-PET) was performed, with the evidence of diffuse hypometabolism in the right frontal brain and right basal nuclei, without any other encephalic area involved. Moreover, diffuse hypermetabolism involving the left ethmoidal and maxillary sinuses was detected, with concomitant hypermetabolic activity in the left axillary and retromandibular lymph nodes (Figure 1). Due to persistent severe thrombocytopenia (platelet count <10,000/mm^3^) requiring repeated platelet transfusions, low-dose corticosteroid therapy was initiated after hematology consultation. However, platelet levels remained critically low despite corticosteroid treatment, necessitating intravenous immunoglobulin infusions (IVIG), escalation of corticosteroid therapy to prednisone 1 mg/kg, and the addition of romiplostim. These interventions ultimately resulted in a platelet count sufficient to safely perform lumbar puncture. On D12 the procedure was performed: on physical–chemical examination, the cerebrospinal fluid was clear and colorless, with a protein concentration of 0.59 g/L, 1 cell/µL, and normal lactate and glucose levels; JCV-DNA (813 cp/mL) and human immunodeficiency virus (HIV)-RNA (552,000 cp/mL) were detected, while Ebstein Barr virus (EBV)-DNA was negative. A repeated encephalic MRI at 26 days showed an increase in the lesion without lesional edema, compatible with evolution of PML in a patient on prednisone 1 mg/Kg therapy for thrombocytopenia of undetermined etiology (Figure 1). Moreover, on D31, considering the presence of hypermethabolic lesions in paranasal sinuses, a biopsy was performed. Since the patient decided to be treated in another hospital center closer to his usual residence, the patient was discharged on D34 with the following blood test results: CD4+ 280/mmc (13.7%), HIV-RNA 1630 cp/mL, serum JCV-DNA detected (<306 cp/mL). At the time of discharge, the patient’s neurological symptoms had improved, with a platelet count over 10.000/mm3 not requiring additional transfusion; the result of biopsy was still in course. After the discharge, the result of the biopsy showed the presence of plasmablastic lymphoma of maxillary sinus (immunophenotype: CD138+, CD38+, c-Myc+, Bcl2+, CD45+/−, CD117−/+).

Hence, in September 2024, he was admitted again in another hospital to undergo radiotherapy sessions. During this period of hospitalization, an additional MRI scan was performed, showing further progression of the brain lesion (Figure 1). At present, clinical follow-up is ongoing. The patient remains clinically stable, with near-complete remission of the symptoms documented at onset. Furthermore, a contrast-enhanced brain MRI performed in September 2025 demonstrated a slight reduction in the lesion (image not available).

We collected 21 cases form the literature in which [^18^F]-FDG-PET imaging was used to characterize PML (Table 1).

## 3. Discussion and Conclusions

In our case report, the most likely diagnosis was PML, based on the suggestive clinical presentation and radiological findings. Positron emission tomography scan imaging was performed primarily to support the differential diagnosis—particularly to distinguish PML from CNS lymphoma—and to avoid an invasive procedure such as lumbar puncture and brain biopsy in a thrombocytopenic patient. The documented hypometabolism of the brain lesion allowed us to reasonably exclude a diagnosis of lymphoma. Moreover, a subsequent lumbar puncture was performed, confirming JC virus positivity in the CSF.

In the literature, PET imaging use in PML is limited to a small number of case reports and series, with a total of 21 cases documented, including the present case (Table 1). These studies suggest that PML is typically associated with reduced [^18^F]-FDG uptake (indicative of hypometabolism) in contrast to healthy brain tissue [5]. Notably, 16 cases (76%) have reported decreased [^18^F]-FDG uptake in the affected regions. However, it should be noted that in cases of PML-IRIS, intralesional hypermetabolism may instead be observed. Consequently, PET imaging may not always be reliably helpful in the differential diagnosis of cerebral lesions in patients with acquired immunodeficiency syndrome [7]. A recent study by C. Mahler et al. proposed the use of [^18^F]-GE-18, a translocator protein radioligand that binds to activated macrophages, microglia, and astrocytes, and demonstrated promising results for detecting IRIS in eight patients with natalizumab-associated PML [8].

Although PET imaging is not considered a standard diagnostic tool for PML, it may nonetheless provide valuable information in selected clinical cases, as illustrated in ours.

**Table 1:** Cases described in the literature of patients with PML in which FDG-PET was used to characterize the lesion.

Authors	Year	Number of Cases	Underlying Disease(s)	CSF JCV-DNA	FDG-PET Findings
Kiyosawa et al. [9]	1988	1	CLL	-	Right cerebral and left cerebellar hypometabolism
Hoffman et al. [10]	1993	1	HIV	-	Slight hypometabolism relative to malignancies but hypermetabolism relative to infections
Mark A. Pierce et al. [11]	1995	2	HIV	-	First patient: Intralesional hypometabolism Second patient: Intralesional hypermetabolism
Heald et al. [12]	1996	2	HIV	-	Hypermetabolism relative to infections and similar metabolism to lymphomas
Ochi et al. [13]	1996	1	ATL	-	Intralesional hypometabolism
O’Doherty et al. [14]	1997	3	HIV	-	Hypometabolism compared to malignant lesions
Mertens et al. [15]	2011	1	Post-transplant	-	Intralesional hypermetabolism
Ashesh et al. [16]	2014	1	MS under treatment with natalizumab	-	Diffuse hypometabolism in the left cerebral hemisphere
Shirai et al. [17]	2014	3	1. HBV-related HCC 2. MALT lymphoma 3. SLE and DM under treatment with azathioprine and CCS	1: - 2: 5080 cp/mL 3: 6230 cp/mL	Intralesional hypometabolism
Ishibashi et al. [18]	2017	1	T-cell lymphoma	73,340 cp/mL	Intralesional hypometabolism
Kamourieh et al. [19]	2017	1	MS under treatment with natalizumab	17,000 cp/mL	Intralesional hypometabolism
Baheerathan et al. [20]	2018	1	MS under treatment with natalizumab	-	Hypometabolism at the level of the dentate nucleus Hypermetabolism at the level of the pons and middle cerebellar peduncle, suggestive of PML-IRIS
Barritt et al. [21]	2022	1	MS under treatment with fingolimod	160 cp/mL	Intralesional hypometabolism
Chiba et al. [7]	2024	1	Mantel cell lymphoma	853 cp/mL	Intralesional hypometabolism
Our case	2025	1	HIV	813 cp/mL	Intralesional hypometabolism

HIV: human immunodeficiency virus; ATL: acute T-cell leukemia; CCS: corticosteroids; CLL: chronic lymphocytic leukemia; cp/mL: copies/milliliters; CSF: cerebrospinal fluid; DM: dermatomyositis; FDG-PET: fluorodeoxyglucose positron emission tomography; HBV: hepatitis B virus; MALT: mucosa-associated lymphoid tissue; HCC: hepatocellular carcinoma; MS: multiple sclerosis; SLE: systemic lupus erythematosus; -: not available.

## Data Availability

The original contributions presented in this study are included in the article. Further inquiries can be directed to the corresponding author.

## Abbreviations

The following abbreviations are used in this manuscript:

PML	progressive multifocal leukoencephalopathy
JCV	polyomavirus JC
CNS	central nervous system
FDG-PET	fluorodeoxyglucose positron emission tomography
MRI	magnetic resonance imaging
CSF	cerebrospinal fluid
HIV	human immunodeficiency virus
IRIS	immune reconstitution inflammatory syndrome
PLT	platelets
IVIG	intravenous immunoglobulin
ATL	acute T-cell leukemia
CCS	corticosteroids
CLL	chronic lymphocytic leukemia
DM	dermatomyositis
HBV	hepatitis B virus
HCC	hepatocellular carcinoma
LCC	chronic lymphocytic leukemia
MS	multiple sclerosis
SLE	systemic lupus erythematosus
MALT	mucosa-associated lymphoid tissue
